# Supplementary material for: Melioidosis–a disease of socioeconomic disadvantage
Source: PLoS Negl Trop Dis. 2021 Jun 21;15(6):e0009544. doi: 10.1371/journal.pntd.0009544 (PMC8248627; doi:10.1371/journal.pntd.0009544)
Supplement: Attachment — Submitted filename: Response to reviewers.docx [file pntd.0009544.s001.docx]

2 June 2021

Dear Dr Poonawala and Dr Wunder,

Thank you for the opportunity to submit a revised version of our manuscript. We would like to thank you and the reviewers for the time that you have taken to review our manuscript and your suggestions for its improvement. Please find below our point-by-point responses to your comments. The document is colour-coded (Editor/reviewer comments in blue, our responses in black) to ease legibility.

Thank you for your manuscript. In addition to the reviewers comments, I had a few comments/questions that I was hoping your team could address. Your manuscript is timely and important - these comments are made with the hope of strengthening it.

1) Have the diagnostic methods/yield for melioidosis changed over the period of your study? Could the decline in mortality over time be due to detection of less sick cases? Are you sure that better treatment is the reason for improved outcomes? Could it be earlier presentation?
**Response:** We thank the Editor for highlighting this issue. As we note in the methods, only culture-confirmed cases were included in this series (lines 127-128) and the rates of bacteraemia remained stable during the study (lines 184-186), so detection of less sick cases is unlikely to explain the decline in mortality.

With earlier identification of sepsis and advances in its care, the decline in mortality is not unexpected. Sepsis-related mortality has fallen globally (Rudd et al, Lancet 2020), in Australia (Kaukonen et al, JAMA 2014) and in cases of melioidosis at other Australian centres (Stephens et al, CCM 2016) during the study period.

Although this was not the focus of the study the reason for the reduction in mortality is likely to be multifactorial. These factors include greater awareness of sepsis in the community leading to earlier presentation, earlier identification of sepsis by clinicians leading to earlier antibiotic therapy and electronic dissemination of melioidosis treatment guidelines leading to earlier prescription of therapy with activity against *B. pseudomallei*. An augmented local aeromedical retrieval network may also have contributed. We have expanded the discussion about this point in line with similar comments from reviewer 3 (lines 305-308). Specific improvements in critical care are likely to have been important as well (see Stephens et al CCM 2016). This was not the focus of the manuscript but is the subject of ongoing study at our centre.

2) How do the outcomes change by SEIFA score deciles within Indigenous Australians? And between Indigenous and non-Indigenous Australians at different deciles? This may help tease apart the interaction between Indigenous and non-Indigenous as well as socio-economic status.
**Response:** We thank the Editor for raising this point and agree that this could be teased apart more. We have generated another figure (figure 4) which we hope demonstrates this point more elegantly. Of course, the multivariate analysis (lines 242-245 and table 6) also helps in determining the relative contributions of potentially contributing factors.

3) I would urge caution in assessing causality from this retrospective study; there are may be confounders that were not assessed. For example chronic lung disease, cancer, immunosuppression were all higher in your urban, non-Indigenous, deciles 2-10; those are all risk factors for worse outcome. Can you include duration of symptoms prior to presenation as a predictor of outcomes?
**Response:** We don’t quite follow the Editor’s line of reasoning here, or have we misunderstood the point he is making? Lung disease, cancer and immunosuppression were, indeed, all more common in non-Indigenous, higher decile patients.

While some of these factors may - intuitively - have be risk factors for a worse outcome, in this study they were not. Looking closely at table 5, the Editor will note that none of the variables he has highlighted (chronic lung disease, cancer, immunosuppression, urban residence, non-Indigenous status, SEIFA deciles 2-10) were risk factors for worse outcome.

Indeed, non-Indigenous patients had a lower mortality than Indigenous patients (11/147 (7.5%) versus 26/174 (14.9%), p=0.04). And patients residing in regions with a SEIFA decile between 2 and 10 had a lower case-fatality rate than those residing in a decile 1 region (11/165 (6.7%) versus 26/156 (16.7%), p=0.005).

We performed multivariate analysis (lines 242-245, table 6) - selecting variables for the model using a typical, pre-specified cut-off of p=0.20 in univariate analysis - to control for the type of confounding factors that the Editor describes, but none of the factors that he has mentioned satisfied this criterion. Indeed, residence in a region with a SEIFA score in decile 2-10 is associated with improved outcomes, this is the central point of the study.

Sorry, if we have misunderstood the point the Editor was trying to raise.

We agree that the duration of symptoms prior to presentation would have been interesting as it would have spoken - at least to some degree - about access to care, which might be hypothesised to contribute to the poorer outcomes seen in the residents of the lowest SEIFA decile, but unfortunately the (predominantly) retrospective nature of the data collection, precluded accurate documentation of this figure. We have added this as a limitation of the study (lines 327-328).

4) Would you be able to provide more context to your results - how do risk factors and outcomes compare with studies of melioidosis in other parts of Australia? Would you be able to use population adjusted rates, especially for discussions of mortality ( I do notice that it was done for incidence). If your study design is unique , could you please highlight in what ways it differs from previous studies?
**Response:** We thank the Editor for raising this point. The risk factors and outcomes are very similar to those reported from Royal Darwin Hospital, which has the highest caseload of melioidosis in the country. The pattern of risk factors is also similar to another Australian study from Townsville which is 347 km to the south of Cairns. We highlight this point now in the revision (lines 281-283, references 11 and 24). However, neither study quantified socioeconomic disadvantage in the manner which we have, our study being the first - to our knowledge - to do so. We have highlighted this in the revised manuscript (lines 275-280).

Population adjusted rates for mortality are probably less helpful for the reader than the case-fatality rate, as there were only 37 deaths in a population of over 250,000 over a 23-year period. Case-fatality rate is the most common measure of mortality in the melioidosis literature and will therefore, we believe, be more helpful for the reader, particularly as the incidence of melioidosis has increased significantly over the duration of the study (which we hypothesise - in another submitted manuscript - is due to expanded construction to the south of the city)

5) 1)  Please shorten your discussion - your data prove the larger theme of your paper quite adequately; a shorter discsussion would definitely strengthen your manuscript. The importance of the social determinants are well known, as are the inequities that Indigenous Australians suffer, and the challenges in reducing them. Would you be able to distil it down to what is relevant to your results? And (most respectfully), please try not to editorialize in your discussion and avoid making broad statements.

**Response:** We thank the Editor for their constructive criticism. We have removed several paragraphs from the discussion in the revision and have also removed sentences that might be construed as Editorial. It is now approximately 25% shorter than in the previous version, even after extra text has been added to address some of the other points that have been raised by the Editor and reviewers (now 1222 words, previously 1644 words). We hope that it now reads better.

In turn, it is difficult to address some of the central themes of the paper without raising issues of social equity and highlighting the continuing plight of many Aboriginal and Torres Strait Islander Australians. To not mention these issues, we would argue, is equally remiss.

Reviewer's Responses to Questions
Key Review Criteria Required for Acceptance?
As you describe the new analyses required for acceptance, please consider the following:

Methods
-Are the objectives of the study clearly articulated with a clear testable hypothesis stated?
-Is the study design appropriate to address the stated objectives?
-Is the population clearly described and appropriate for the hypothesis being tested?
-Is the sample size sufficient to ensure adequate power to address the hypothesis being tested?
-Were correct statistical analysis used to support conclusions?
-Are there concerns about ethical or regulatory requirements being met?

Reviewer #1: Yes

Reviewer #2: Are the objectives of the study clearly articulated with a clear testable hypothesis stated?
Yes
-Is the study design appropriate to address the stated objectives?
Yes
-Is the population clearly described and appropriate for the hypothesis being tested?
Yes
-Is the sample size sufficient to ensure adequate power to address the hypothesis being tested?
Yes
-Were correct statistical analysis used to support conclusions?
Yes
-Are there concerns about ethical or regulatory requirements being met?
No

Reviewer #3: No major concerns. The study methods are well-described and appropriate.

**Response:** We are heartened by the fact that the reviewers were happy with the methods that we employed.

Results
-Does the analysis presented match the analysis plan?
-Are the results clearly and completely presented?
-Are the figures (Tables, Images) of sufficient quality for clarity?

Reviewer #1: Yes

Reviewer #2: Does the analysis presented match the analysis plan?
Yes
-Are the results clearly and completely presented?
Yes
-Are the figures (Tables, Images) of sufficient quality for clarity?
Yes

Reviewer #3: No concerns. The results are clearly presented.
**Response:** We are happy to see that the reviewers have no concerns with the results.

Conclusions
-Are the conclusions supported by the data presented?
-Are the limitations of analysis clearly described?
-Do the authors discuss how these data can be helpful to advance our understanding of the topic under study?
-Is public health relevance addressed?

Reviewer #1: Yes

Reviewer #2: Are the conclusions supported by the data presented?
Yes
-Are the limitations of analysis clearly described?
Yes
-Do the authors discuss how these data can be helpful to advance our understanding of the topic under study?
Yes
-Is public health relevance addressed?
Yes

Reviewer #3: Again, no concerns. The conclusions are supported by the data and the discussion is very appropriate.

**Response:** We are heartened by the fact that the reviewers were happy with our discussion and conclusions.

Editorial and Data Presentation Modifications?
Use this section for editorial suggestions as well as relatively minor modifications of existing data that would enhance clarity. If the only modifications needed are minor and/or editorial, you may wish to recommend “Minor Revision” or “Accept”

Reviewer #1: (No Response)

Reviewer #2: (No Response)

Reviewer #3: This is a well designed study with suitable analysis and presentation of results.
I have only a few minor comments:
It would be interesting to see a brief presentation and discussion of any data relating to delays in access to care for subjects in the study e.g. distance (space and time) from health facilities, or time to diagnosis of melioidosis after presentation. In other countries, the time to access medical care has an influence on mortality rates.
Urban/rural and remote might be better defined in the Methods - e.g. would Innisfail, Palm Cove or Port Douglas count as rural areas?
Could the authors discuss the possible causes for the declining case-fatality rate over the course of the study, and how this might relate to socioeconomic disadvantage?
**Response:** We are pleased that reviewers 1 and 2 are happy with the paper. We agree that discussing time to presentation would have allowed us to examine issues around access to care. However, as we have discussed in reply to a similar comment to the Editor, it was not possible to retrieve reliable data for this variable in this retrospective study. Charts were destroyed or documentation was inadequate in many cases. We have added this as a limitation of our study in the revised manuscript (lines 327-328).

Definitions of urban and rural/remote were based on the author’s assessment. Inevitably there may be some grey areas in this definition, but we contend that they would have very little effect on the findings of the study. With regard to the specific locations highlighted by the reviewer, Innisfail would have been defined as rural/remote, but there were no cases in this town. Palm Cove (1 case) and Port Douglas (1 case) are both are popular - somewhat exclusive - beachside resort towns; both were classified as “urban” in this study.

Presenting cases by individual town would have little relevance to the Journal’s International readership; but more importantly, our ethics approval precluded us identifying individual towns or communities, given the socioeconomic issues that we were discussing, due to concerns about stigmatisation.

We have presented some - speculative - hypotheses for why the case-fatality rate may have been declining over the study period (lines 305-308). As highlighted above, this is the basis of ongoing research at our centre.
--------------------

Summary and General Comments
Use  this section to provide overall comments, discuss strengths/weaknesses of the study, novelty, significance, general execution and scholarship. You may also include additional comments for the author, including concerns about dual publication, research ethics, or publication ethics. If requesting major revision, please articulate the new experiments that are needed.

Reviewer #1: This is a well written and interesting paper that demonstrates a clear association between indicators of population disadvantage and both incidence and mortality from melioidosis.  My only questions are whether the authors would be able to include any individual-specific data about socio-economic status and disadvantage, and also whether it would be possible to include an analysis of duration of symptoms prior to admission to hospital as a possible explanation for the observed mortality difference?
**Response:** As highlighted above, our ethics approval precluded us identifying individual towns or communities, given the socioeconomic issues that we were discussing, due to concerns about stigmatisation. We have also addressed the duration of symptoms issue in previous responses.

Reviewer #2: There is little information about the socioeconomic impact on melioidosis. This paper revealed that socioeconomically disadvantaged patients were more likely to have melioidosis and associated with mortality. The study suggests a holistic approach to the delivery of healthcare which addresses the social determinants of health is important, to reduce the burden of melioidosis as well as other diseases.
The topic is important and relevance to neglected tropical infections like melioidosis. It will be of interest for policy maker and researches. The conclusion is justified based on the current data. The study raises the questions whether this finding would be true in other counties.
**Response:** We thank Reviewer 2 for their generous comments.

Reviewer #3: This is a good study, which will be of considerable relevance for public health management of melioidosis (and other infectious diseases). **Response:** We thank Reviewer 3 for their generous comments.
